# Role of airway glucose in bacterial infections in patients with chronic obstructive pulmonary disease

**DOI:** 10.1016/j.jaci.2017.10.017

**Published:** 2018-09

**Authors:** Patrick Mallia, Jessica Webber, Simren K. Gill, Maria-Belen Trujillo-Torralbo, Maria Adelaide Calderazzo, Lydia Finney, Eteri Bakhsoliani, Hugo Farne, Aran Singanayagam, Joseph Footitt, Richard Hewitt, Tatiana Kebadze, Julia Aniscenko, Vijay Padmanaban, Philip L. Molyneaux, Ian M. Adcock, Peter J. Barnes, Kazihuro Ito, Sarah L. Elkin, Onn Min Kon, William O. Cookson, Miriam F. Moffat, Sebastian L. Johnston, John S. Tregoning

**Affiliations:** a Airway Disease Infection Section, National Heart and Lung Institute, Imperial College, London, United Kingdom; e Airways Disease Section, National Heart and Lung Institute, Imperial College, London, United Kingdom; f Molecular Genetics and Genomics Section, National Heart and Lung Institute, Imperial College, London, United Kingdom; b Imperial College Healthcare, National Health Service Trust, London, United Kingdom; c Cardiff University School of Medicine, UHW Main Building Heath Park Cardiff, Cardiff, United Kingdom; d Mucosal Infection and Immunity Group, Section of Virology, Imperial College London, St Mary's Campus, London, United Kingdom

**Keywords:** Chronic obstructive pulmonary disease, glucose, viral infection, airway inflammation, bacterial infection, ASL, Airway surface liquid, BAL, Bronchoalveolar lavage, COPD, Chronic obstructive pulmonary disease, DTT, Dithiothreitol, GOLD, Global Initiative for Obstructive Lung Disease, NL, Nasal lavage

## Abstract

**Background:**

Patients with chronic obstructive pulmonary disease (COPD) have increased susceptibility to respiratory tract infection, which contributes to disease progression and mortality, but mechanisms of increased susceptibility to infection remain unclear.

**Objectives:**

The aim of this study was to determine whether glucose concentrations were increased in airway samples (nasal lavage fluid, sputum, and bronchoalveolar lavage fluid) from patients with stable COPD and to determine the effects of viral infection on sputum glucose concentrations and how airway glucose concentrations relate to bacterial infection.

**Methods:**

We measured glucose concentrations in airway samples collected from patients with stable COPD and smokers and nonsmokers with normal lung function. Glucose concentrations were measured in patients with experimentally induced COPD exacerbations, and these results were validated in patients with naturally acquired COPD exacerbations. Relationships between sputum glucose concentrations, inflammatory markers, and bacterial load were examined.

**Results:**

Sputum glucose concentrations were significantly higher in patients with stable COPD compared with those in control subjects without COPD. In both experimental virus-induced and naturally acquired COPD exacerbations, sputum and nasal lavage fluid glucose concentrations were increased over baseline values. There were significant correlations between sputum glucose concentrations and sputum inflammatory markers, viral load, and bacterial load. Airway samples with higher glucose concentrations supported more *Pseudomonas aeruginosa* growth *in vitro*.

**Conclusions:**

Airway glucose concentrations are increased in patients with stable COPD and further increased during COPD exacerbations. Increased airway glucose concentrations might contribute to bacterial infections in both patients with stable and those with exacerbated COPD. This has important implications for the development of nonantibiotic therapeutic strategies for the prevention or treatment of bacterial infection in patients with COPD.

Chronic obstructive pulmonary disease (COPD) is the third leading cause of death worldwide.^1^ COPD is characterized by an abnormal pulmonary inflammatory response after exposure to noxious gases, such as cigarette smoke. Airway inflammation persists even after smoking cessation, and therefore other factors, such as infection, contribute to the persistence of inflammation and disease progression. The clinical course of COPD is characterized by a progressive worsening of exercise tolerance and health status punctuated by periods of increased symptoms termed acute exacerbations. Exacerbations are a major cause of morbidity and mortality in patients with COPD and are associated with a rapid decrease in lung function, increased inflammation, and impaired quality of life.^2^ Bacterial infections are detected in 30% to 40% of patients with stable COPD and in 50% of patients with acute COPD exacerbations.^2^ Susceptibility to respiratory tract infection is increased in patients with COPD, but the mechanisms are poorly understood, with most research focusing on deficiencies in immune responses.^2^, ^3^ Changes in nutrient concentrations in the airways that are required for bacterial growth, such as glucose, might also increase susceptibility to infection.

Glucose concentrations in airway surface liquid (ASL) are normally 12 times lower than blood glucose concentrations, and this might be a homeostatic mechanism inhibiting bacterial growth by depriving the bacteria of an essential nutrient.^4^, ^5^ *In vitro* and animal studies demonstrate that increased ASL glucose concentrations promote bacterial lung infection. *Staphylococcus aureus* and *Pseudomonas aeruginosa* use glucose as a growth substrate, and their growth is promoted by high ASL glucose concentrations.^6^, ^7^, ^8^, ^9^ There is a link between increased airway glucose concentrations and bacterial colonization in animal models,^8^, ^10^, ^11^ but the relationship between airway glucose concentrations and infection *in vivo* in human subjects is less well studied. One study has linked glucose in bronchial aspirates to infection with methicillin-resistant *S aureus*^12^; however, sputum glucose concentrations were not related to exacerbation frequency in patients with cystic fibrosis.^13^

Airway inflammation in *in vitro* models increases ASL glucose concentrations, likely because of increased epithelial permeability and glucose flux.^14^, ^15^ Because there is chronic airway inflammation in patients with COPD, this could result in increased airway glucose concentrations in patients with COPD.^4^ However, to date, no studies have investigated airway glucose concentrations and their relationship with airway inflammation and infection in patients with COPD.

We have developed a human infection challenge model of COPD exacerbation using experimental rhinovirus infection that allows for collection of multiple samples during exacerbations under carefully controlled conditions and have demonstrated roles for viral and bacterial infection, inflammation, and oxidative stress in patients with COPD exacerbations.^16^, ^17^, ^18^ Therefore this model provides a tool with which to examine relationships between infection, inflammation, and airway glucose concentrations. We hypothesized that airway glucose concentrations are increased in patients with COPD and further increased in patients with COPD exacerbations when airway inflammation is greater and that glucose concentrations are related to airway inflammatory markers and bacterial infection. We used airway samples from experimental rhinovirus infection studies to investigate these hypotheses. We then repeated the analyses in samples collected from a separate cohort of patients with COPD with naturally acquired exacerbations to validate our results from experimental infection studies.

## Methods

### Study participants

The samples used were collected from subjects recruited to 2 previously published experimental infection studies and from a cohort of patients with COPD with naturally acquired exacerbations.

#### Stable COPD

The stable COPD samples were baseline samples of subjects from the experimental infection studies^17^ and the stable time point samples from the naturally acquired exacerbation cohort. Subjects in the experimental infection studies who were not successfully infected were not included in the exacerbation results, but their baseline samples were used for analysis of stable COPD.^17^ All stable samples were collected when subjects had been free of infection and had been treated with antibiotics or oral corticosteroids for at least 8 weeks.

#### Experimental infection studies

Inclusion criteria and data from the subjects in the experimental rhinovirus infection studies have been published previously and are described in the Methods section in this article's Online Repository at www.jacionline.org.^16^, ^17^, ^18^ Three groups of subjects were recruited: patients with COPD (Global Initiative for Obstructive Lung Disease [GOLD] stage II), smokers with normal lung function, and healthy nonsmokers. Patients with COPD were allowed to use short-acting bronchodilators only. All subjects provided written informed consent, and the study protocol was approved by St Mary's NHS Trust Research Ethics Committee (study nos. 00/BA/459E and 07/H0712/138). Baseline samples of nasal lavage (NL) fluid, sputum, and bronchoalveolar lavage (BAL) fluid were obtained before infection, subjects were inoculated with rhinovirus 16, and postinfection samples were collected, as described previously.^16^, ^18^

#### Naturally acquired COPD exacerbation cohort

A separate cohort of 40 patients with COPD was recruited in which all grades of COPD severity and all treatments were permitted. Subjects provided written informed consent, and the study protocol was approved by the East London Research Ethics Committee (study no. 11/LO/0229). NL fluid and sputum were collected at baseline, 3-month intervals, exacerbation onset, and 2 and 6 weeks after exacerbation. Further clinical details for this cohort are provided in the Methods section in this article's Online Repository.

#### Measurement of glucose

Glucose was measured by using the Amplex Red Glucose Assay Kit (Invitrogen, Thermo Fisher Scientific, East Grinstead, United Kingdom). Standards and samples were prepared, according to the manufacturer's instructions; fluorescence was read on a microplate reader; and results were analyzed by using SoftMax Pro software (Molecular Devices, Sunnyvale, Calif). Further details are provided in the Methods section in this article's Online Repository. Blood glucose concentrations were measured in the clinical biochemistry laboratory of Imperial College Healthcare NHS Trust.

#### Inflammatory mediators

The Meso Scale Discovery platform (Meso Scale Discovery, Rockville, Maryland) was used to measure inflammatory mediators, according to the manufacturers' instructions, as published previously.^16^ The human proinflammatory 4-plex kit was used to measure IL-6, IL-1β, IL-8 (CXCL8), and TNF levels. Further details are provided in the Methods section in this article's Online Repository.

#### Bacterial and virological analyses

Respiratory tract viruses were detected by using standard PCR assays, as described previously, and bacteria were cultured in the microbiology laboratory of Imperial College Healthcare NHS Trust.^17^ Rhinovirus load was measured by using quantitative PCR. For measurement of 16s RNA, genomic DNA was extracted from sputum according to a modified protocol provided with the QIAamp DNA Mini Kit (Qiagen, Manchester, United Kingdom). The V3-V5 region of the bacterial 16S rRNA gene was then amplified and quantified by using the 357F forward primer and the 926R reverse primer, as previously described.^19^

### *In vitro P aeruginosa* growth

One hundred microliters of sputum or nasal samples were inoculated with 2 × 10^5^ colony-forming units of log-phase *P aeruginosa* (strain PAO1) and incubated for 4 hours at 37°C and 200 rpm on 96-well plates before determining OD_600_ with a FLUOstar Omega Microplate Reader.

### Statistical analysis

The clinical characteristics of the subjects are presented as means. All study data are presented as medians, and changes from baseline were analyzed by using the Friedman test and Dunn multiple comparisons test. Between-group differences were analyzed by using the Kruskal-Wallis test and Dunn multiple comparisons test. Correlations between data sets were examined by using the Spearman rank correlation coefficient. Because samples were collected on multiple time points in the experimental infection studies, peak postinfection values for each subject were used to examine correlations. Differences were considered significant for all statistical tests at *P* values of less than .05. Analysis was performed with GraphPad Prism software for Windows (version 6.00; GraphPad Software, La Jolla, Calif).

## Results

### Study participants

The clinical characteristics of the subjects included in this study are shown in Table I. Five subjects had a clinical diagnosis of type 2 diabetes mellitus (1 smoker and 4 patients with COPD). Three were diet controlled, and 2 were taking oral hypoglycemic medications (metformin). FEV_1_ was significantly less in the patients with COPD compared with values in smokers and nonsmokers; there was no difference in lung function between smokers and nonsmokers.

**Table I:** Clinical characteristics of study subjects

	Subjects included in stable analysis			Subjects experimentally infected with rhinovirus			Naturally acquired exacerbation study cohort	
	NS (n = 19)	SMK (n = 29)	COPD (n = 77)	NS (n = 10)	SMK (n = 15)	COPD (n = 15)	Total (n = 40)	Subjects reporting an exacerbation (n = 17)
Age (y)	58.95 ± 1.59	52.10 ± 1.41	63.27 ± 1.12	62.2 ± 1.79	49.73 ± 2.05	60.13 ± 2.21	66.39 ± 1.53	67.00 ± 2.58
Sex (male/female)	9/10	17/12	53/24	4/6	6/9	8/7	28/12	12/5
Smoking history (pack years)	0	33.41 ± 1.77	48.68 ± 2.80	0	32.40 ± 2.36	43.47 ± 5.19	53.02 ± 4.39	55.35 ± 8.05
FEV_1_ (L)	3.056 ± 0.22	3.27 ± 0.12	1.89 ± 0.08	2.7 ± 0.18	3.140 ± 0.20	1.9 ± 0.12	1.75 ± 0.12	1.79 ± 0.21
FEV_1_ (% predicted)	106 ± 3.79	102 ± 2.48	65.56 ± 1.83∗	100.8 ± 3.31	99.13 ± 3.2	66.93 ± 1.5∗	63.50 ± 3.13	62.4 ± 5.13
FEV_1_/FVC ratio	78.96 ± 0.93	77.43 ± 1.04	54.07 ± 1.44	77.98 ± 1.34	78.83 ± 1.47	58.68 ± 2.19	50.06 ± 2.26	50.12 ± 3.18
GOLD stage (I/II/III/IV)	NA	NA	11/56/6/4	NA	NA	0/15/0/0	8/22/6/4	3/10/2/2
Diabetes (treatment)	0	1 (diet)	4 (2 diet, 2 OHA)	0	0	1 (OHA)	3 (2 diet, 1 OHA)	2 (1 diet, 1 OHA)
Treatment								
No treatment	19	29	12	10	15	7	5	3
SAB	0	0	15	0	0	8	7	1
LAB	0	0	8	0	0	0	8	4
ICS ± LAB	0	0	20	0	0	0	20	9

All data are presented as means ± SEMs.

*FVC*, Forced vital capacity; *ICS*, inhaled corticosteroids; *LAB*, long-acting bronchodilators; *NA*, not applicable; *NS*, nonsmokers; *OHA*, oral hypoglycemic agent; *SAB*, short-acting bronchodilators; *SMK*, smokers.

∗ *P* < .0001 versus smokers and nonsmokers.

From the experimental infection studies, samples were available from 15 patients with COPD, 15 smokers, and 10 nonsmokers. Patients with COPD with naturally occurring exacerbations reported 27 exacerbations. Infections were detected in 24 (89%) of these exacerbations: viral infection, 67%; bacterial infection, 22%; dual viral-bacterial infection, 15%; and no infection, 11%. None of the experimental infection exacerbations were treated. Five (18.5%) of 27 of the naturally acquired exacerbations were treated with oral corticosteroids, antibiotics, or both, but samples were collected before initiation of treatment.

### Airway glucose concentrations in patients with stable COPD

**Fig 1:**
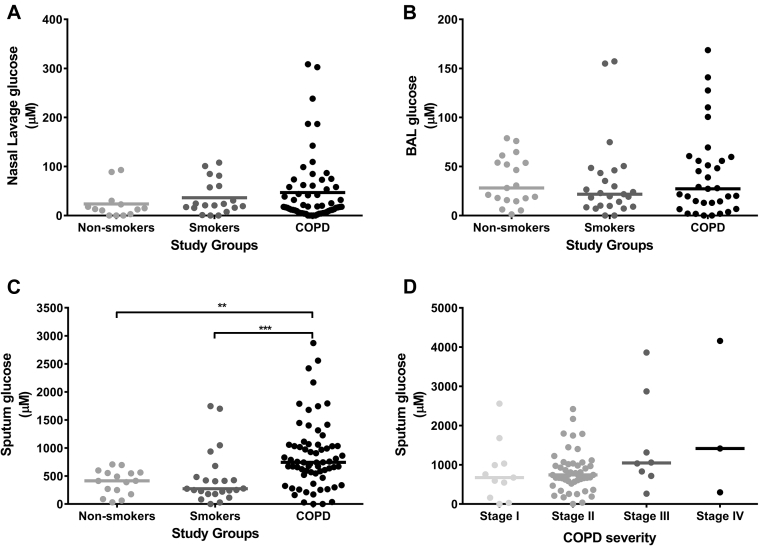
Airway glucose concentrations in stable subjects. All data are shown as medians. **A,** NL fluid glucose concentrations. **B,** BAL fluid glucose concentrations. **C,** Sputum glucose concentrations. **D,** Sputum glucose concentrations according to GOLD stages. **P < .01 and ***P < .001.

**Fig E1:**
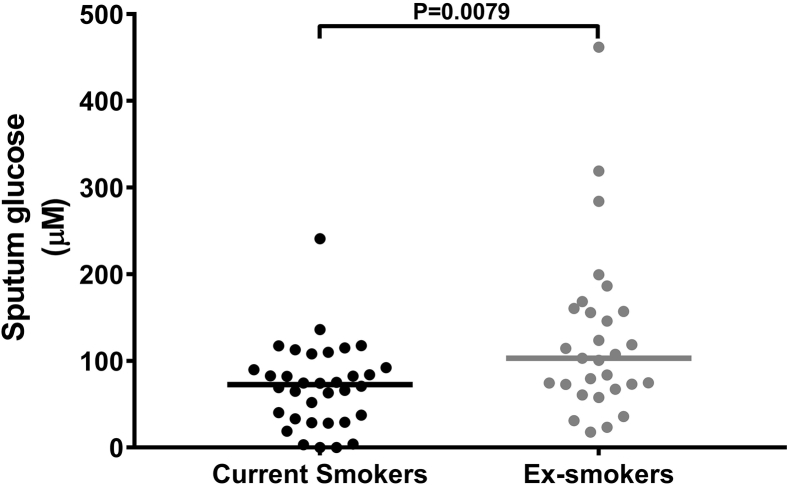
Sputum glucose concentrations according to smoking status in patients with COPD.

There were no differences in glucose concentrations in NL or BAL fluid between patients with COPD and control subjects without COPD (Fig 1, *A* and *B*), but sputum glucose concentrations were significantly greater in patients with COPD compared with those in smokers and nonsmokers (Fig 1, *C*). These differences remained significant if subjects with diabetes were excluded (data not shown). There were no differences between the smokers and nonsmokers. There was a trend toward progressively higher sputum concentrations of glucose from GOLD stage I (673 μmol/L; interquartile range, 160.9-1030 μmol/L) to GOLD stage IV (1414 μmol/L; interquartile range, 298.3-4158 μmol/L), but this was not statistically significant (Fig 1, *D*). There was a negative correlation between sputum glucose concentrations and FEV_1_ in all subjects combined (*P* < .0001, *r* = −0.39). Sputum glucose concentrations in patients with COPD were significantly higher in ex-smokers compared with those in current smokers (see Fig E1 in this article's Online Repository at www.jacionline.org).

### Airway glucose concentrations in patients with COPD exacerbations

**Fig 2:**
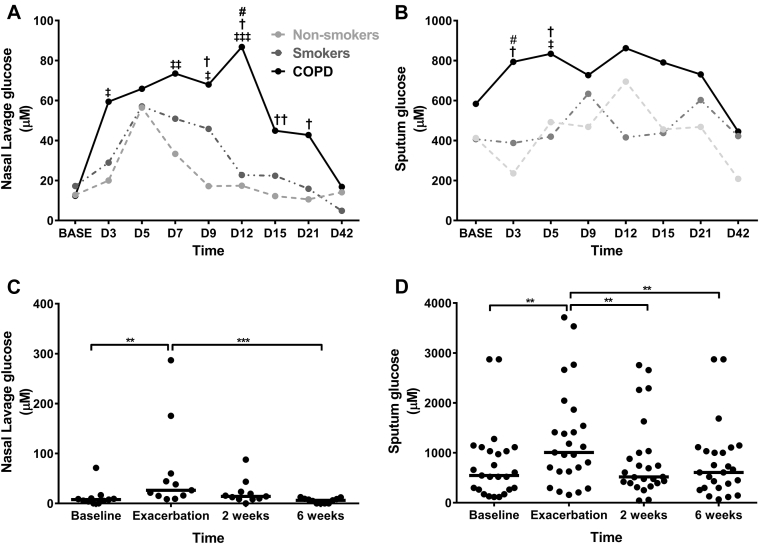
Airway glucose concentrations in patients with COPD exacerbations. All data are shown as medians. **A,** NL fluid glucose concentrations in subjects experimentally infected with rhinovirus. **B,** Sputum glucose concentrations in subjects experimentally infected with rhinovirus. **C,** NL fluid glucose concentrations in patients with naturally acquired COPD exacerbations. **D,** Sputum glucose concentrations in patients with naturally acquired COPD exacerbations. **P < .01 and ***P < .001, †*P* < .05 versus nonsmokers, ††*P* < .01 versus nonsmokers, ‡*P* < .05 versus baseline, ‡‡*P* < .01 versus baseline, ‡‡‡*P* < .001 versus baseline, and #*P* < .05 versus smokers.

There were significant increases from baseline values in NL fluid glucose concentrations after experimental rhinovirus infection in patients with COPD (Fig 2, *A*). NL fluid glucose concentrations were significantly higher in the COPD group compared with nonsmokers on days 9, 12, 15, and 21 and on day 12 compared with those in smokers. Sputum glucose concentrations were also increased significantly after infection in patients with COPD and were higher compared with those in nonsmokers on days 3 and 5 and on day 3 compared with those in smokers (Fig 2, *B*). There were no significant increases in BAL fluid glucose concentrations after infection. In the patients with naturally acquired COPD exacerbations, NL fluid glucose concentrations increased significantly at exacerbation compared with baseline and 6 weeks (Fig 2, *C*), and sputum glucose concentrations significantly increased at exacerbation compared with those at baseline, 2 weeks, and 6 weeks (Fig 2, *D*).

### Airway glucose concentrations, inflammation, and viral load

**Fig 3:**
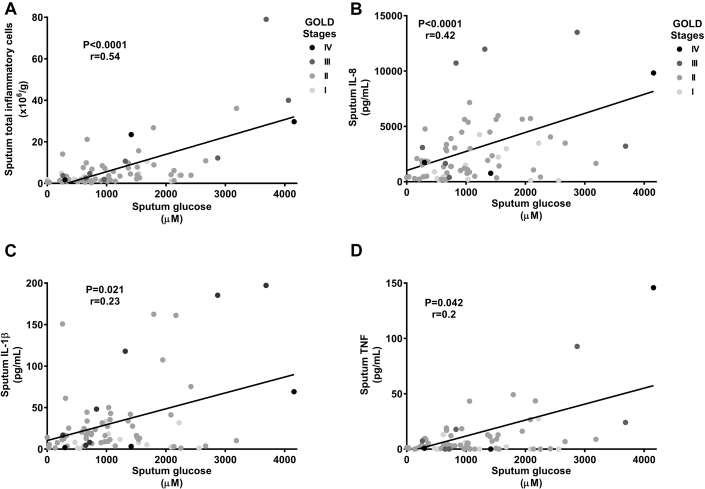
Correlations between sputum glucose concentrations and levels of inflammatory markers in patients with stable COPD. **A,** Sputum total inflammatory cell numbers. **B,** Sputum IL-1β levels. **C,** Sputum IL-8 levels. **D,** Sputum TNF levels.

In patients with stable COPD, there were significant correlations between glucose concentrations and total sputum inflammatory cell counts (Fig 3, *A*), sputum IL-1β levels (Fig 3, *B*), IL-8 levels (Fig 3, *C*), and TNF levels (Fig 3, *D*). In patients with rhinovirus-induced COPD exacerbations, there were significant correlations between peak postinfection sputum glucose concentrations and peak sputum inflammatory cell counts (*P* = .013, *r* = 0.57), sputum IL-β levels (*P* < .0001, *r* = 0.87), IL-8 levels (*P* = .0018, *r* = 0.73), and TNF levels (*P* = .0018, *r* = 0.73).

**Fig 4:**
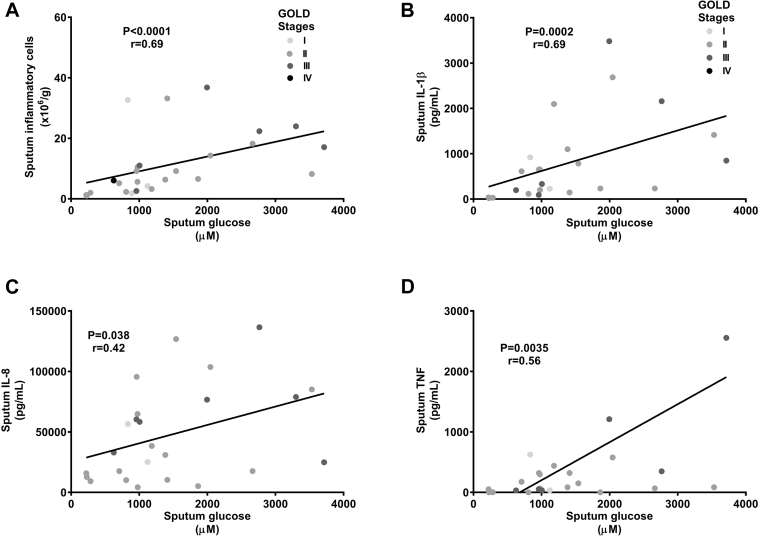
Correlations between sputum glucose concentrations and inflammatory markers in patients with naturally acquired COPD exacerbations. **A,** Sputum total inflammatory cell numbers. **B,** Sputum IL-1β levels. **C,** Sputum IL-8 levels. **D,** Sputum TNF levels.

**Fig 5:**
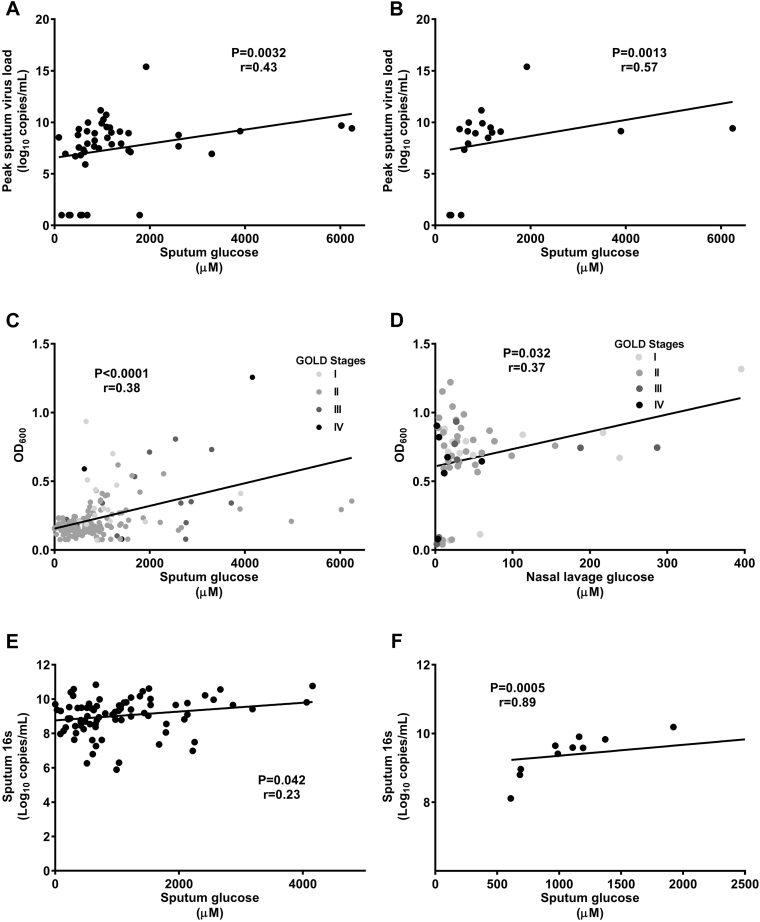
Correlations between sputum glucose concentrations and viral and bacterial loads. **A,** Peak sputum viral load in all subjects with experimental rhinovirus infections. **B,** Peak sputum viral load in patients with COPD and experimental rhinovirus infections. **C,***In vitro* bacterial growth in sputum. **D,***In vitro* bacterial growth in NL fluid. **E,** Bacterial 16s expression in stable samples. **F,** Day 15 rhinovirus postinfection bacterial 16s expression.

Correlations between sputum glucose concentrations and levels of inflammatory markers in patients with naturally acquired exacerbations are shown in Fig 4. Natural acquired exacerbation was associated with an increase in neutrophils recovered from the sputum (see Fig E4 in this article's Online Repository at www.jacionline.org). In patients with rhinovirus-induced COPD exacerbations, there were significant correlations between peak sputum viral load and peak sputum glucose concentrations in all subjects combined (Fig 5, *A*) and in patients with COPD (Fig 5, *B*).

### Airway glucose and bacterial load

**Fig E2:**
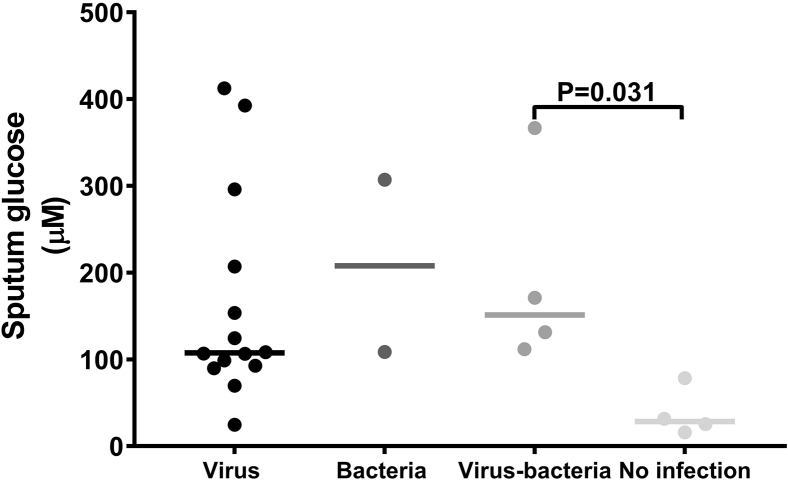
Sputum glucose concentrations in patients with naturally acquired exacerbations according to exacerbation cause.

**Fig E3:**
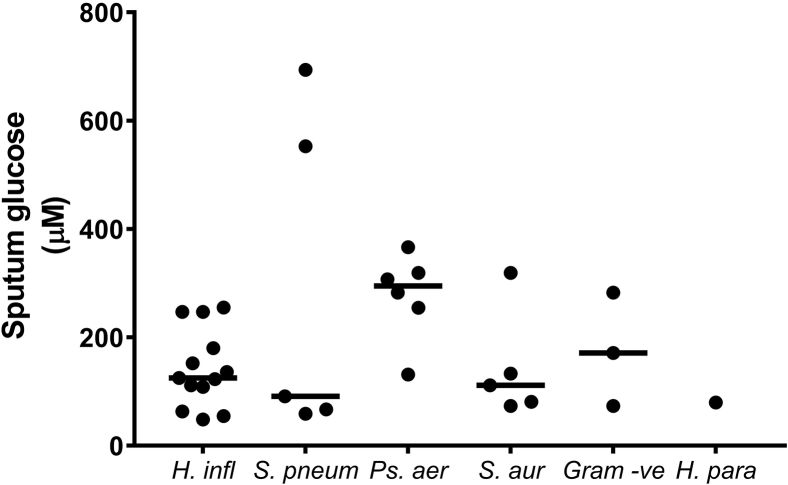
Sputum glucose concentrations and bacterial species. Gram–ve, Gram negative; *H. infl*, *Haemophilus influenzae*; *H. para*, *Haemophilus parainfluenzae*; *Ps. aer*, *Pseudomonas aeruginosa*; *S. aur*, *Staphylococcus aureus*; *S. pneum*, *Streptococcus pneumoniae*.

**Fig E4:**
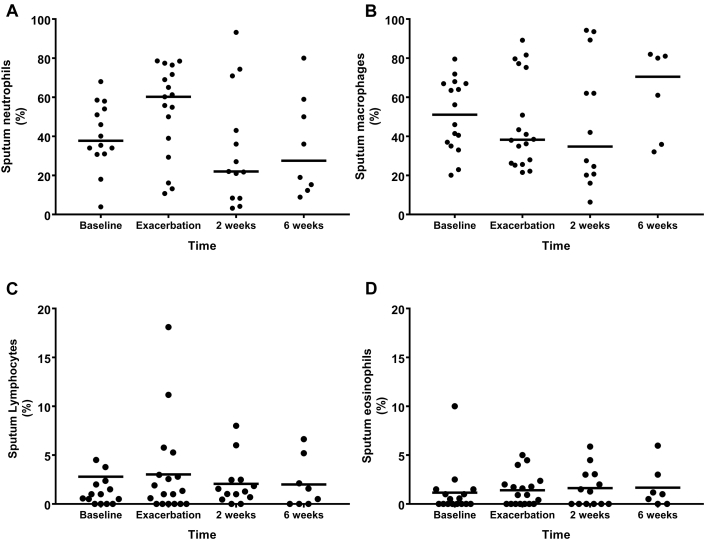
Differential cell counts in patients with naturally acquired exacerbations. **A,** Percentage of neutrophils in sputum. **B,** Percentage of macrophages in sputum. **C,** Percentage of lymphocytes in sputum. **D,** Percentage of eosinophils in sputum.

*In vitro P aeruginosa* growth in sputum or NL fluid correlated with sputum (Fig 5, *C*) or NL fluid (Fig 5, *D*) glucose concentrations, respectively. There was no correlation with *P aeruginosa* growth and BAL fluid glucose concentrations. In stable subjects there was a weak but significant correlation between sputum bacterial 16s rRNA and glucose concentrations (Fig 5, *E*). Peak postinfection sputum glucose concentrations correlated significantly with day 15 sputum 16s rRNA (Fig 5, *F*). Sputum glucose concentrations in patients with naturally acquired exacerbations did not differ significantly between exacerbations with a viral, bacterial, or dual cause. However sputum glucose concentrations were significantly greater in patients with dual-cause exacerbations when compared with those with exacerbations in which no infection was detected (see Fig E2 in this article's Online Repository at www.jacionline.org). There were no differences in sputum glucose concentrations between exacerbations with different bacterial species (see Fig E3 in this article's Online Repository at www.jacionline.org).

### Airway and blood glucose concentrations

There was a significant but weak correlation between NL fluid and sputum glucose concentrations (*P* = .0005, *r* = 0.19), but there were no significant relationships between sputum and NL fluid glucose concentrations with BAL fluid glucose concentrations. In a subset of subjects (19 nonsmokers, 13 smokers, and 20 patients with COPD), nonfasting blood glucose values were available. All were within the normal range (<7.8 mmol/L), and there was no significant relationship between blood and airway glucose concentrations.

## Discussion

This is the first study measuring glucose concentrations in airway samples from patients with COPD and examining relationships between airway glucose concentrations, inflammation, and infection. We demonstrate that airway glucose concentrations are increased in both patients with stable COPD and those with COPD exacerbations and report relationships between airway glucose concentrations, inflammation, and bacterial load. These results are seen in both patients with experimentally induced and those with naturally acquired COPD exacerbations.

Glucose concentrations have been measured previously in airway samples, including sputum,^13^ exhaled breath condensate,^15^ nasal secretions,^6^ and bronchial aspirates,^12^ by using different methods and in different patient groups. Because the optimum sample type was not known, we measured glucose concentrations in NL fluid, sputum, and BAL fluid. Glucose concentrations in BAL fluid were generally low, did not correlate with sputum or NL values, and were not increased in patients with stable COPD or after viral infection. BAL fluid is affected by variable degrees of dilution, and we have reported similar results with inflammatory mediators.^18^ In view of this variability and because measurements of inflammatory mediators and bacterial and virus load were available in sputum, we focused our analysis on sputum glucose concentrations as a measure of lower airways glucose concentrations.

The median sputum glucose concentration was 390.5 μmol/L in control subjects without COPD and 743 μmol/L in patients with stable COPD. By means of comparison, median sputum glucose concentrations in patients with cystic fibrosis is 700 μmol/L (although using different methods of sputum processing and glucose detection), and therefore airway glucose concentrations in patients with COPD are comparable with those reported in patients with cystic fibrosis.^13^ Sputum glucose concentrations were significantly greater in patients with COPD compared with those in both healthy smokers and nonsmokers, with a trend toward higher sputum glucose concentrations with higher GOLD stage and a negative relationship with FEV_1_, suggesting a relationship with COPD severity. However, numbers of patients with COPD in GOLD stages III and IV were small.

Respiratory tract viruses are a common cause of COPD exacerbations,^20^ and bacterial infections often occur after viral infection.^19^, ^21^ Viral infections increase airway inflammation,^16^, ^18^ but other than a study of nasal glucose in symptomatic colds,^22^ the effects of viral infection on airway glucose concentrations are unknown. After rhinovirus infection, there were significant increases in glucose concentrations in both upper and lower airway samples in patients with COPD but not in control subjects without COPD, with airway glucose concentrations returning to baseline levels with exacerbation resolution.

To validate these results, we then measured airway glucose concentrations in a separate cohort of patients with COPD experiencing naturally acquired exacerbations. We reported a similar pattern of increased airway glucose concentrations at exacerbation with a return to baseline values 6 weeks after exacerbation.

This is the first report of increased airway glucose concentrations in both patients with stable COPD and those with COPD exacerbations. Increased glucose concentrations have been associated with multiple adverse effects, including impaired epithelial healing,^23^ airway hyperresponsiveness,^24^ neutrophil activation,^25^ immune suppression,^26^ and enhanced growth of bacterial pathogens.^7^, ^12^ Therefore the airway glucose concentration is a possible mechanism contributing to the pathogenesis of COPD and COPD exacerbations and a potential therapeutic target.

Airway glucose concentrations are increased by airway inflammation or hyperglycemia. Only 5% of subjects were diabetic, and blood glucose concentrations were normal in a sample of 52 subjects. Therefore increased airway glucose concentrations in the presence of normoglycemia are likely related to increased leakage of glucose across an inflamed epithelium. This has been described *in vitro*,^14^ but the relationship between airway inflammation and glucose concentrations *in vivo* has not been examined. The significant correlations we report between sputum inflammatory markers and glucose concentrations in patients with stable COPD, with even stronger correlations in exacerbation samples, support an association between airway inflammation and airway glucose concentrations. Therefore these are the first *in vivo* human data linking airway inflammation to airway glucose concentrations in patients with COPD, particularly in patients with COPD exacerbations.

An association between airway glucose concentrations and increased susceptibility to bacterial infection has been proposed from studies in animals and patients with cystic fibrosis^4^ but has not been investigated in patients with COPD. Bacterial infections occur after viral infection in both patients with experimentally induced^17^, ^19^ and those with naturally acquired^21^ rhinovirus infections, and we hypothesized that this might be linked to changes in airway glucose concentrations. When *P aeruginosa* was cultured *in vitro* on sputum and NL fluid samples from our subjects, those samples with higher glucose concentrations supported greater growth of *P aeruginosa*. Examining the relationships between sputum glucose concentrations and bacterial load *in vivo*, there was a weak but significant correlation between bacterial 16s rRNA and sputum glucose concentrations in patients with stable COPD. Previously, we reported that secondary bacterial infections occurred in 60% of patients with rhinovirus-induced COPD exacerbations, with bacterial load peaking on day 15 after rhinovirus infection.^17^, ^19^ In the exacerbation samples from the experimental rhinovirus studies, there was a strong correlation between peak postinfection sputum glucose concentrations and day 15 sputum 16s rRNA expression. Therefore together these data provide the first *in vivo* evidence linking airway glucose concentrations to enhanced bacterial growth in patients with COPD.

These data have a number of implications for the treatment of COPD and other pulmonary diseases. The increasing detection of gram-negative organisms displaying antimicrobial resistance in patients with COPD highlights the need for novel nonantibiotic therapies.^27^, ^28^ Treatments that reduce airway glucose concentrations might have potential as nonantibiotic treatments to prevent bacterial infections in patients with COPD.^4^ In animal models the antihyperglycemic agents metformin and dapagliflozin reduced airway glucose concentrations and inhibited growth of *S aureus* or *P aeruginosa*.^8^, ^10^, ^11^ However, a recent trial of metformin in hospitalized patients with COPD found no effect on inflammatory mediators or clinical outcomes.^29^ In this study metformin was commenced after hospital admission, with the full dose reached after only 4 days. Our data demonstrate that airway glucose concentrations and inflammatory mediators peak soon after exacerbation onset, and therefore metformin might have been given too little and too late to be effective in reducing airway glucose concentrations sufficiently to produce a significant clinical effect. An alternative strategy that warrants investigation is long-term use of metformin to reduce airway glucose in patients with stable COPD, thereby potentially inhibiting bacterial growth and reducing both primary and postviral bacterial exacerbations.

Inflammation and bacterial infection play a prominent role in other pulmonary diseases, including asthma,^30^ bronchiectasis,^31^ and pulmonary fibrosis.^32^ Increased airway glucose concentrations can contribute to susceptibility to respiratory tract infection in these diseases, and further studies investigating this are warranted.

Our study has a number of limitations. The majority of the patients with COPD (76%) were GOLD stage II, and the prevalence of diabetes was low; therefore patients with more severe and diabetic COPD were underrepresented. However, it is likely that the prevalence and effect of increased airway glucose concentrations is even greater in these groups, and therefore our results might be even more relevant to such patients. Further studies with larger subject numbers and a wider range of COPD severity are needed. We used *P aeruginosa* to investigate relationships between bacterial growth and glucose concentrations, but it is not known whether this is relevant to other pathogens, such as *Haemophilus influenzae*, which are more prevalent in patients with COPD. Finally, only glucose was measured, but there are other metabolites in the airways that influence bacterial growth.

Our data should stimulate further studies into this novel area, investigating the links between the airway metabolic profile, inflammation, and infection. Investigating the effects of interventions that reduce airway inflammation on airway glucose concentrations and airway infection will be key to understanding these relationships.

In conclusion, our data demonstrate that airway glucose concentrations are increased in patients with stable COPD and patients with COPD exacerbations. Airway glucose concentrations and airway inflammation are related, and enhanced bacterial growth is associated with increased airway glucose concentrations. We propose a sequence of events whereby airway inflammation increases airway glucose concentrations in patients with COPD contributing to chronic bacterial infection. Acute viral infection induces an acute airway inflammatory response, thereby further increasing glucose leakage into the airways and promoting secondary bacterial infection. Therapeutic interventions that reduce airway glucose concentrations might have potential as nonantibiotic therapies for preventing bacterial infection in patients with COPD.

**Key messages**

• Sputum glucose concentrations are increased in patients with stable COPD and virus-induced COPD exacerbations.
• Airway glucose concentrations in patients with COPD are related to bacterial infection, but the direction of the causal relationship is uncertain.
• Therapeutic strategies to reduce airway glucose concentrations might provide a nonantibiotic method to reduce or prevent bacterial infections in patients with COPD.

## Methods

### Experimental rhinovirus infection

The samples used in this study were collected from 2 experimental rhinovirus infection studies. Clinical and laboratory data from these studies have been published previously.E1, E2 Inclusion/exclusion criteria are listed below. Baseline samples of induced sputum, NL fluid, and BAL fluid were obtained approximately 14 days before infection, and subjects were inoculated with 10 median tissue culture infective dose of rhinovirus 16 on day 0. Nasal and sputum sampling were repeated on subsequent visits on days 3, 5, 9, 12, 15, 21, and 42 after infection and bronchoscopy on day 7.

### Inclusion and exclusion criteria for experimental infection studies

#### All subjects

• Age 40-75 years
• No history of asthma or allergic rhinitis and not atopic on skin testing
• Absence of a current or previous history of bronchiectasis, carcinoma of the bronchus, or other significant respiratory disease (other than COPD)
• Absence of significant systemic disease
• No COPD exacerbation or respiratory tract infection within the previous 8 weeks
• Serum antibodies to rhinovirus 16 at screening in a titer of less than 1:2
• No treatment with antibiotics; oral, inhaled, or nasal topical steroids; long-acting β-agonists; or long-acting antimuscarinics in the previous 3 months

#### COPD group

• FEV_1_ of 50% to 79% of predicted normal value and β-agonist reversibility of less than 12%
• FEV_1_/FVC ratio of less than 70%
• Current smokers or ex-smokers with at least 20 pack years of cumulative smoking

#### Smokers

• FEV_1_ of 80% or greater of predicted normal value
• FEV_1_/FVC ratio of greater than 70%
• Current smokers or ex-smokers with at least 20 pack years of cumulative smoking

#### Nonsmokers

• FEV_1_ of 80% or greater of predicted normal value
• FEV_1_/FVC ratio of greater than 70%
• Nonsmokers

### Study subjects

Clinical data from the experimental infection studies have been published previously.E1, E2, E3 Data from patients with naturally acquired infections are unpublished. These subjects were a cohort of 40 patients with COPD, the clinical characteristics of whom are shown in Table I. Only subjects with GOLD stage II COPD were included in the experimental infection studies, whereas subjects with disease of all severities were recruited in the other COPD cohort. Subjects in experimental infection studies were allowed use of short-acting bronchodilators only, whereas all treatments were allowed in the naturally acquired infection cohort. No subjects were receiving long-term antibiotics or phosphodiesterase E4 inhibitors. Five subjects had a diagnosis of type 2 diabetes, 3 of whom were diet controlled and 2 of whom were taking metformin.

### Exacerbations

In experimental infection studies subjects kept a daily symptom diary card, and therefore a COPD exacerbation was defined as an increase in lower respiratory score of at least 2 points over baseline for at least 2 consecutive days, according to our previous studies.E1, E2, E3 In the naturally acquired infection study a clinical definition of an exacerbation was used based on the GOLD definition (ie, “acute worsening of the patient's respiratory symptoms that was beyond normal day-to-day variations and may require a change in medication”). Twenty-seven exacerbations were reported by 17 subjects, and 23 subjects reported no exacerbations. Subjects received treatment (oral corticosteroids or antibiotics or both) for 13 (48%) exacerbations, and 14 (52%) exacerbations were not treated.

### Clinical samples

The methods of collecting, processing, and analyzing clinical samples were the same in all studies and have been published previously.E1, E2

### Induced sputum

Sputum was induced and processed by using protocols used in our previous studies.E1, E2 Subjects were premedicated with 200 μg of salbutamol through a metered-dose inhaler and large-volume spacer, and baseline FEV_1_ was measured. Four percent saline was administered with a DeVilbiss UltraNeb99 ultrasonic nebulizer (DeVilbiss, Somerset, Pa) until an adequate sputum sample was obtained. Sputum was processed within 2 hours of induction. Sputum plugs were selected from saliva by means of macroscopic inspection of the sample. An aliquot was selected and stored unprocessed at −80°C for quantitative RT-PCR for viral load. The remaining sample was weighed, 0.1% dithiothreitol (DTT) was added in a ratio of 4 mL of DTT to 1 g of sputum, and the mixture was agitated and filtered. The same volume of PBS was added, the filtrate was centrifuged, and the supernatant was placed in aliquots and stored at −80°C. The cell pellet was washed and resuspended, and cells were counted to obtain total cell counts. Cytospin preparations were prepared and stained with the Shandon DiffQuick kit (Thermo Shandon, Cheshire, United Kingdom), coded, and counted blind to study status to obtain differential cell counts. Cell counts were expressed as a percentage of at least 400 inflammatory cells.

### Nasal lavage

NL was performed by instilling 2.5 mL of 0.9% saline into each nostril, holding for 5 seconds, and then expelling into a sterile container. The lavage fluid obtained was homogenized, placed in aliquots, and stored at −80°C.

### BAL

An aliquot of unfiltered BAL fluid was stored for quantitative RT-PCR for viral load, the remaining BAL fluid was filtered and centrifuged, and the supernatant was stored.

### Meso Scale Discovery

The technique enables quantitative detection of mediators in a 96-well plate format by using a Multi-spot technique. This technology uses a capture antibody attached to each spot within the well, which enables measurement of multiple mediators simultaneously. Electrochemiluminescence of detection antibody is then recorded with an internal high-sensitivity camera (SECTOR Imager; Meso Scale Discovery). Briefly, the protocol requires addition of 25 μL of blocking solution before incubation. After plate washing, either sample or standard was added to the plate, followed by incubation and washing and addition of detection antibody. Finally, read buffer was added, and the plate was passed through the SECTOR Imager for reading. The lower limits of detection of the individual analytes were as follows: IL-1β, 1.17 pg/mL; CXCL8/IL-8, 0.6 pg/mL; and TNF-α, 0.376 pg/mL.

### Glucose detection

Glucose concentrations were measured with the Amplex Red Glucose Assay Kit (Invitrogen, Thermo Fisher Scientific). Standards and samples were prepared according to the manufacturer's instructions. The Amplex Red Glucose/Glucose Oxidase Assay Kit (A22189) provides a sensitive 1-step method for detecting glucose in bodily fluids. All samples were analyzed in duplicate, and a standard curve was generated for each plate. Fluorescence was read on a microplate reader, and results were analyzed with SoftMax Pro software. Because DTT can interfere with the Amplex Red Glucose Assay, initial experiments were carried out analyzing glucose concentrations in sputum samples processed with and without DTT. Detection of glucose was consistently more reliable, and higher concentrations were detected in sputum processed with DTT; therefore these samples were used to carry out the analysis.

## Results

### Sputum glucose concentrations and smoking status

Sputum glucose concentrations in patients with stable COPD were significantly greater in ex-smokers compared with those in current smokers (Fig E1).

### Sputum glucose and exacerbation cause

Sputum glucose concentrations in patients with naturally acquired exacerbations were analyzed according to exacerbation cause. There were no differences in sputum glucose concentrations between viral, bacterial, and dual viral-bacterial exacerbations. Sputum glucose concentrations in exacerbations in which dual viral-bacterial infection was present were greater compared with those in patients with exacerbations but in whom no infection was detected (Fig E2). There were no differences in sputum glucose concentrations between exacerbation samples with different bacterial species cultured (Fig E3).

### Differential sputum cell counts

Differential cell counts from experimental rhinovirus infection studies have been published previously.E1, E2 Fig E4 shows differential cell counts in patients with naturally acquired COPD exacerbations. We examined the relationships between differential cell counts and sputum glucose concentrations. There were no significant correlations between sputum glucose concentrations and cell differentials (macrophages, neutrophils, lymphocytes, or eosinophils) in either the stable samples, samples from the naturally acquired infections, or samples from the experimental infections.
